# Novel piRNA Regulates PIWIL1 to Modulate the Behavior of Placental Trophoblast Cells and Participates in Preeclampsia

**DOI:** 10.1155/2022/7856290

**Published:** 2022-04-14

**Authors:** Jing Lin, Ye Zhou, Wei Gu

**Affiliations:** ^1^International Peace Maternity and Child Health Hospital, School of Medicine, Shanghai Jiao Tong University, 910 Hengshan Road, Shanghai 200030, China; ^2^Shanghai Municipal Key Clinical Specialty, Shanghai, China

## Abstract

**Objectives:**

This study is aimed at investigating the role of PIWIL1/piRNA in the development of preeclampsia.

**Methods:**

High-throughput sequencing was performed in 5 preeclampsia and 5 normal placentas to get a piRNA expression profile. WGCNA network was constructed to find hub piRNAs. Through target gene prediction and protein interaction network analysis, we found the potential relationship between the key genes and PIWIL1. Subsequently, we detected the expression of PIWIL1 in 35 preeclampsia and 29 normal placental tissues. Overexpression and inhibition of PIWIL1 in HTR-8/SVneo trophoblast cells were achieved by transfecting an overexpression vector and siRNAs, respectively. Cell proliferation, apoptosis, and invasion were assessed using CCK-8, flow cytometric, and transwell assays, respectively.

**Results:**

It was found that a total of three piRNAs were upregulated in preeclampsia (pir-hsa-1256314, uniq_271431, and uniq_277797). And two target genes with the highest connectivity (FXR1 and DDX6) both pointed to PIWIL1. PIWIL1 expression was significantly lower in preeclampsia. In vitro studies linked PIWIL1 expression to trophoblast overgrowth. Overexpression of PIWIL1 remarkably promoted cell proliferation and invasion and inhibited apoptosis of HTR-8/SVneo cells and vice versa.

**Conclusions:**

PIWIL1/piRNA may be involved in the pathogenesis of preeclampsia by inhibiting the proliferation and invasion and promoting the apoptosis of placental trophoblasts. This study was registered with the China Clinical Trials Registry (http://www.clinicaltrials.gov): registration number ChiCTR1900027479.

## 1. Introduction

Preeclampsia is an idiopathic disease of pregnancy occurring after 20 weeks of pregnancy, involving multiple organs and seriously affecting the health of pregnant women and perinatal infants [1, 2]. The incidence rate of preeclampsia is 2%~8% [1, 2]. The incidence of preeclampsia is 2%–8% [3, 4]. Preeclampsia still has no effective treatments other than delivery, and symptomatic medications simply slow illness development [4]. Therefore, we need to explore the potential pathogenesis of preeclampsia. The clinical symptoms of most patients with preeclampsia are quickly relieved and improved after a pregnancy is terminated, indicating that the placenta plays a vital role in the pathogenesis of preeclampsia [1, 5]. Placental trophoblast cells, which are the main cells in the placenta, function in invasion and vascular recast and maintain the normal development of the placenta [6, 7]. It is widely believed that the pathological origin of preeclampsia is related to the abnormal function of placental trophoblasts and inadequate vascular recast [8]. Excessive apoptosis and dysfunction of trophoblast cells can cause shallow placental implantation and incomplete uterine vascular recast, which affect the exchange of oxygen and nutrients between mother and fetus, leading to chronic hypoxia of the placenta and induction of pregnancy-related diseases, such as preeclampsia and fetal intrauterine growth restriction [9, 10].

PIWI proteins are important members of the Argonaute family, which are predominantly expressed in the germline [11]. PIWI proteins mainly interact with PIWI-interacting RNAs (piRNAs), which leads to transposon gene silencing and protects the stability and integrity of the genome in germline cells [11]. Increasing evidence has linked PIWI/piRNA to tumorigenesis [12], neurodevelopmental and neurodegenerative diseases [13], and diabetes and cardiovascular diseases [14]. As an important member of the PIWI family, PIWI-like protein 1 (PIWIL1) has been reported to be abnormally expressed in many cancers and to mediate various cytological processes, such as cell proliferation, apoptosis, and invasion as well as cell-cycle arrest [15, 16]. However, the role of PIWIL1/piRNA in preeclampsia has not been investigated. In this study, we obtained the differentially expressed piRNA between preeclampsia and normal pregnant women through high-throughput sequencing and determined their target genes. It is found that these differentially expressed piRNAs are closely related to PIWIL1. Therefore, we speculated that PIWIL1/piRNA plays a vital role in the development of preeclampsia. We further investigated the role of PIWIL1 by overexpressing and silencing its expression in vitro to obtain evidence for the association of PIWIL1/piRNA with the development and progression of preeclampsia. This study took the similarity between embryo implantation and tumor invasion as the starting point and explored whether the abnormal expression of PIWI/piRNA in the placenta is involved in regulating trophoblast cell invasion. It provides a new research direction for explaining the pathogenesis of preeclampsia, offers potential targets for its prediction and treatment.

## 2. Methods

### 2.1. Patients and Sample Acquisition

**Figure 1:**
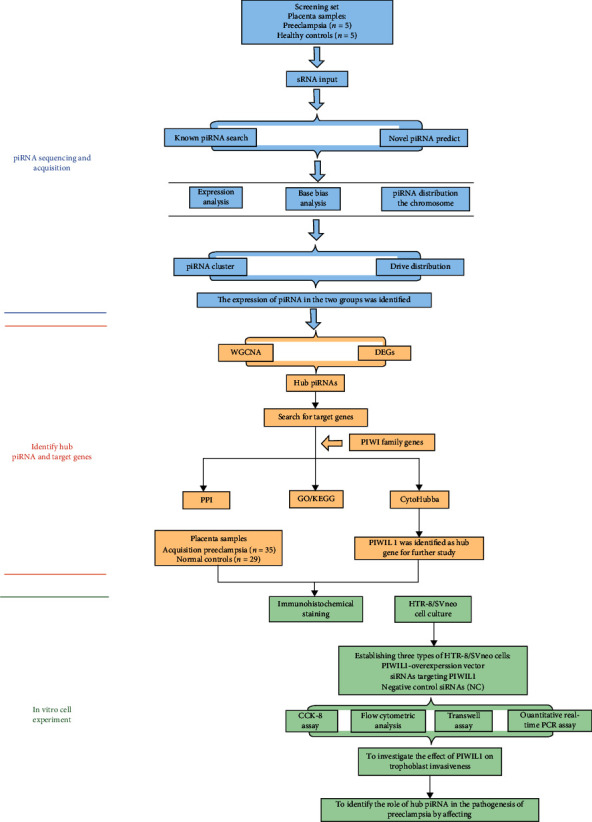
Flowchart of study design.

From January 2021 to June 2021, 40 patients with preeclampsia and 34 patients with normal pregnancies (controls) who underwent a cesarean section in the Obstetrics Department of the International Peace Maternity and Child Health Hospital were enrolled in this study. Patients were excluded if they had gestational diabetes or other pregnancy complications, renal disease, multiple pregnancies, thyroid dysfunction, or chronic hypertension. We collected placental tissue samples from all enrolled individuals. The placental tissue was taken from the area near the umbilical cord insertion site and collected rapidly within 30 minutes after cesarean section. These tissue samples were cut into small pieces using sterile scissors, washed with sterile PBS. A portion was snap-frozen in liquid nitrogen and then stored in RNAlater reagent (Thermo Fisher Scientific, USA) at −80°C for RNA extraction and western blotting, and a portion was fixed with formalin and embedded in paraffin for immunohistochemical staining. In this experiment, 5 preeclampsia and 5 normal placental tissues were used for high-throughput sequencing, and the remaining 35 disease groups and 29 control groups were used as the validation of the cell experiment in vitro. The flowchart in Figure 1 illustrates the whole process of this study. This study was approved by the ethics committee of the International Peace Maternal and Child Health Hospital (Approval No. GKLW 2021-23) and has been registered in the Chinese clinical trial registry (http://www.clinicaltrials.gov; registration number: ChiCTR1900027479), and all patients provided written informed consent.

### 2.2. High-Throughput Sequencing

RNA sequencing: samples of 5 cases of preeclampsia and 5 cases of normal placental cells were sent to Shanghai ERYUN Information Technology Co., Ltd. for high-throughput sequencing. MicroRNAs were constructed and sequenced by the Illumina HiSeq3000 platform. The HTSeq software was used to make statistics on the original miRNA deep sequencing data. After screening and processing, the reads less than 15 nt and greater than 41 nt were screened to obtain clean reads. Noncoding RNAs were annotated as rRNAs, tRNAs, snRNAs, and snoRNAs. By comparing the piRBase v1.0 database, we identified known piRNAs and predicted new piRNAs by analyzing uncommented small RNAs.

### 2.3. Construction of Weighted Gene Coexpression Network and Identification of Significant Modules

The data were processed using the R-Studio 3.4.0 software. To ensure that the results of network construction were reliable, abnormal samples were removed. The WGCNA package was used to construct the coexpression network. First, the samples were clustered to assess the presence of any significant outliers. Second, the coexpression network is constructed using the automatic network construction function, which uses the R function to pick up the soft threshold to calculate the soft threshold setting power *β*. The similarity of coexpressions is proposed to calculate the adjacency. Hierarchical clustering is performed on each block to produce a tree diagram, and the modules are designated as branches. Automatic module merging was performed for modules with highly correlated feature piRNAs (max block size = 4000, T0M type = unsigned, min module size = 20, and merge cut height = 0.2). As a result, piRNAs with similar expression patterns were grouped into a single module, and each such module was given a color. Module affiliation (MM) and gene significance (GS) were calculated for modules associated with clinical attributes. The piRNA information in the modules is used for further analysis. Finally, the characteristic piRNA network was visualized. The connection between the piRNA module and clinical characteristics is extremely reliable if the piRNAs in a module show a high correlation between module membership and piRNA importance (association with traits). To this end, we mapped the membership and piRNA significance of the modules related to clinical traits.

### 2.4. Identification of Differentially Expressed Genes and Prediction of Target Genes

The screening standard of differentially expressed genes was *p* < 0.05 and the differential multiple was ≥2. Three tools such as TargetScan (http://www.targetscan.org), miRDB-MicroRNA Target Prediction Database (http://mirdb.org), and MrmicroT (http://diana.imis.athena-innovation.gr/DianaTools/index.php?r=mrmicrot/index) were used to predict the target genes of these target piRNAs.

### 2.5. Functional Enrichment Analysis and KEGG Analysis

The target genes were uploaded to DAVID 6.8 (http://david-d.ncifcrf.gov/) for functional enrichment analysis. Gene ontology (GO) analysis is used to determine differentiating biological characteristics. To determine functional attributes, the Kyoto Encyclopedia of Genes and Genomes (KEGG) pathway enrichment analysis was used.

### 2.6. Protein-Protein Interaction (PPI) Network Construction

The previously acquired genes from each module are mapped to the search platform STRING database (STRING, V11.0; https://string-db.org/) to comprehensively identify the hub genes of each module and the distinctive genes of the modules. It is essential in the protein-protein network (PPI). The CytoHubba plug-in is based on the Cytoscape software (http://www.cytoscape.org/, version 3.7.1; Institute for Systems Biology, Seattle, WA, USA), which was then used to construct and visualize the protein interactions of 66 target genes and four types of PIWI.

### 2.7. Cell Culture

The human extravillous trophoblast cell line HTR-8/SVneo and the choriocarcinoma cell lines JAR and JEG-3 were purchased from Procell Life Science & Technology Co., Ltd. All cells were maintained in Ham's F 12 medium (Hyclone) supplemented with 10% heat-inactivated FBS and 1% penicillin-streptomycin at 37°C in a 5% CO_2_ incubator.

### 2.8. Cell Transfection

HTR-8/SVneo cells were seeded into 6-well plates and grown until 80% confluence for transfection. The PIWIL1-overexpression vector, small interfering RNAs (siRNAs) targeting PIWIL1, and the corresponding negative control siRNA (NC) were transfected into HTR-8/SVneo cells using Lipofectamine 2000 (Invitrogen) according to the manufacturer's protocol. The sequences of the siRNAs are shown in Table 1. After 48 h of transfection, the cells were harvested for subsequent experiments.

### 2.9. Cell Proliferation Assay

The proliferation ability of trophoblast cells was detected by the CCK-8 method. HTR-8/SVneo cells in the logarithmic growth phase were digested with trypsin (Genom Biomedical Technology Co., Ltd.) and then resuspended at a concentration of 1 × 105 cells/mL. The cells were seeded in 96-well plates (100 *μ*l/well) and incubated overnight at 37°C. Then, the medium was changed to serum-free medium supplemented with 10 *μ*L of CCK-8 solution (Beyotime) and incubated in the dark for 2 h. After treatment, the absorption at 450 nm was determined with a microplate reader (DR-200Bs; Diatek).

### 2.10. Flow Cytometric Analysis

HTR-8/SVneo cells were seeded at 3 × 105 cells/well in a six-well plate and incubated overnight. Then, cell apoptosis was assessed using the Annexin V-FITC/PI kit (Tianjin Sungene Biotech Co., Ltd.) according to the manufacturer's instructions. Briefly, cells were resuspended in 300 *μ*L of binding buffer, and 5 *μ*l of FITC-Annexin-V-FITC was added, and the plate was incubated in the dark for 10 min. Then, 5 *μ*l of PI was added, and the plate was incubated in the dark for 5 min. Finally, the cells were analyzed for apoptosis using a flow cytometer (BD Aria III).

### 2.11. Transwell Assay

Cell invasion was evaluated using a Matrigel transwell assay (Corning). In brief, after 24 h of transfection, HTR-8/SVneo cells were harvested and resuspended in a serum-free medium. Then, 200 *μ*L of the cell suspension was placed into the upper chamber, and a complete medium containing 10% FBS was added to the lower chamber. After incubation at room temperature for 48 h, the invaded cells in the lower chamber were fixed with 4% paraformaldehyde for 20 min and then stained with 0.1% crystal violet (Wuhan Aspen Biotechnology) for 10 min. The number of cells that invaded the lower chamber was counted under an inverted microscope (IX51; Olympus).

### 2.12. Quantitative Real-Time PCR Assay

Total RNA was isolated with TRIzol reagent and was dissolved in 10 *μ*L of RNase-free water. Complementary DNA (cDNA) was synthesized utilizing the EntiLink™ 1st Strand cDNA Synthesis Kit (ELK Biotechnology), and real-time qPCR was conducted to assess gene expression utilizing EnTurbo™ SYBR Green PCR SuperMix (ELK Biotechnology) on a StepOne™ Real-time PCR system (Life Technologies) under the following thermal cycling conditions is as follows: 95°C for 3 min, followed by 40 cycles at 95°C for 10 s, 58°C for 30 s, and 72°C for 30 s. Relative expression was evaluated using the 2-*ΔΔ*Ct method. The sequences of the primers used were as follows: PIWIL1 sense 5′-ACTAACTCCAGAGCAAAGGCAG-3′ and antisense 5′-CCTTGGTGAATCTTTTCTGTTTG-3′, and GAPDH (reference gene) sense 5′-CATCATCCCTGCCTCTACTGG-3′ and antisense 5′-GTGGGTGTCGCTGTTGAAGTC-3′.

### 2.13. Western Blotting

Placental tissues or HTR-8/SVneo cells were lysed with RIPA lysis buffer (ASPEN) in the presence of protease inhibitor, and then, the isolated protein was quantified using the BCA Protein Assay Kit (ASPEN). Proteins were separated using SDS-PAGE and transferred onto methanol-activated PVDF membranes. The membranes were then blocked with skim milk and incubated with an anti-PIWIL1 primary antibody (Abcam # ab181056) at 4°C overnight. After washing with PBST buffer, the membrane was incubated with the secondary antibody (ASPEN # AS1107) for 30 min. The bound antibodies were detected using High-sig ECL Substrate. The gray value of the protein bands was analyzed using the ImageJ software.

### 2.14. Statistical Analysis

Data are presented as the mean ± standard deviation and were analyzed using SPSS 21.0 (IBM, USA). Two groups were compared using the *t*-test, and three groups were compared using the one-way ANOVA followed by Dunnett's multiple tests. Statistical significance was set at *p* < 0.05.

## 3. Result

### 3.1. Clinical Characteristics

The clinical characteristics of the enrolled individuals are listed in Table 2. Compared with the normal group, the preeclampsia group had earlier gestational weeks, lower birth weight, lower Apgar score, and higher incidence of postpartum hemorrhage.

### 3.2. Sequence Signature and Genomic Mapping of piRNAs in Human Placenta

**Figure 2:**
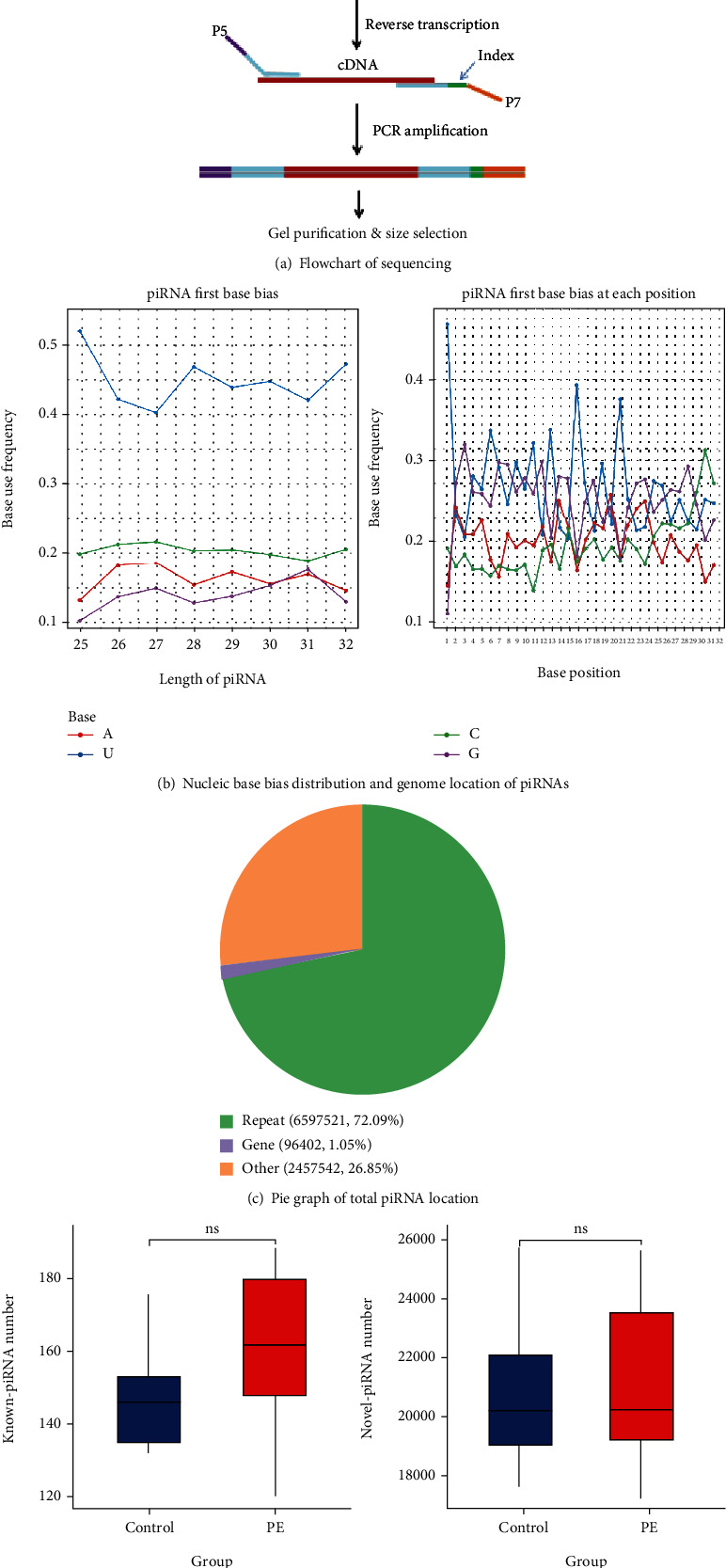
(a) Flowchart of sample detection, database establishment, and sequencing. (b) Visual diagram of preference of the first base of piRNAs with different lengths. Visual diagram of base bias at each site of the piRNA sequence. (c) Pie graph of total piRNA location in the genome. (d) Numbers of putative piRNAs in both groups.

piRNA-seq was performed in 5 preeclampsia and 5 control placentas (Figure 2(a)). In our data, the sequence fragment had a significant main peak at 26-32 nt, which is the characteristic length of piRNAs. And like miRNA, the 5′ terminal also has an obvious preference for U, and some bases at position 10 also have a preference for A (Figure 2(b)). piRNA is not evenly distributed on different chromosomes and is not proportional to the length of chromosomes. piRNA obtained was compared with transposon sequence and gene sequence in order to explore the distribution of piRNA on the genome. Most piRNAs are located in the repetition zone, as shown in Figure 2(c). The piRNA sequences obtained were compared with those recorded in the database, and the total piRNAs were divided into known piRNAs and novel-defined piRNAs (Figure 2(d)). There was no significant difference in the number of piRNAs between the two groups.

### 3.3. Construction of Weighted Coexpression Network and Identification of Key Modules

**Figure 3:**
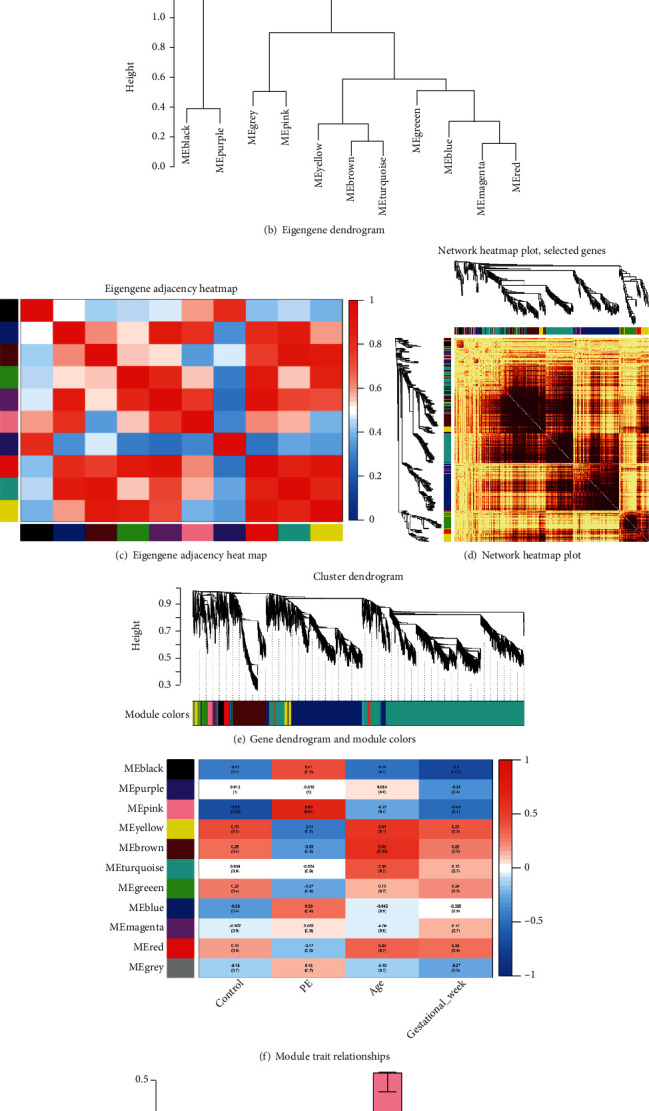
(a) Analysis of network topology for different soft-thresholding powers. The left panel shows the influence of soft-thresholding power (*x*-axis) on the scale-free fit index (*y*-axis). The right panel displays the influence of soft-thresholding power (*x*-axis) on the mean connectivity (degree, *y*-axis). (b) An eigengene dendrogram identified groups of correlated modules. (c) Eigengene adjacency heatmap of different gene coexpression modules. (d) Interaction of coexpression genes based on TOM dissimilarity and the cluster dendrogram of 1,000 randomly selected genes. The colors of the axes represent respective modules. The intensity of the yellow inside the heatmap represents the overlap degree of overlap, with a darker yellow representing an increased overlap. (e) Cluster dendrogram. Each color represents one specific coexpression module, and branches above represent genes. (f) Module-trait relationship heatmap for different traits and gene modules. Values in the figure indicate the correlation coefficient between modules and clinical traits. Values in brackets are the *p* values for the association test. (g) Barplot of mean gene significance across modules. In this example, we use a trait-based gene significance. The higher the mean gene significance in a module, the more significantly related the module is to the clinical trait of interest.

The expression matrix of sequenced piRNA (182139 piRNAs) was obtained. After data preprocessing, we filtered the missing value or low expression objects (6171 piRNAs). Then, we selected the piRNAs with MAD > 1 and in the top 5000. The samples were clustered by Pearson correlation coefficient, and we set the soft threshold to 6 (*R*^2^ = 0.9) to construct a scale-free network. One-step network construction and module detection were performed. Next, we constructed an adjacency matrix and a topological overlap matrix (Figure 3). Finally, 11 modules were identified based on average hierarchical clustering and dynamic tree clipping. The pink module was highly correlated with the onset of preeclampsia (*p* = 0.04) and was the only module with statistical significance (Figure 3(f)). Barplot of mean gene significance across modules showed a trait-based gene significance. The higher the mean gene significance in a module, the more significantly related the module is to the clinical trait of interest. Among them, the gene significance of pink module is the highest (Figure 3(g)). Therefore, pink module genes are most associated with the onset of preeclampsia, and it was selected as a clinically meaningful module for further analysis.

### 3.4. Screening Differentially Expressed Genes (DEGs) and Determination of Target piRNAs

**Figure 4:**
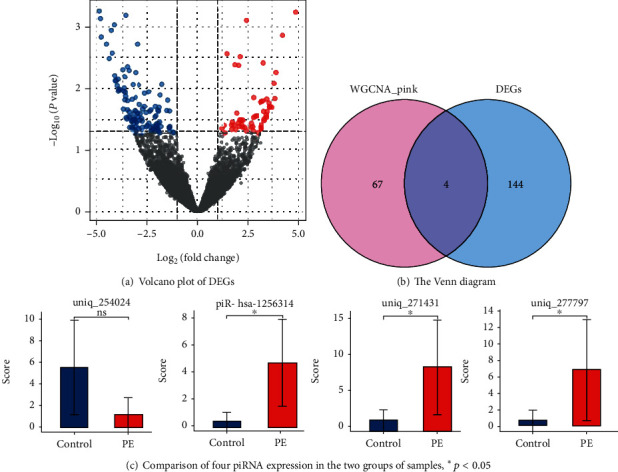
(a) Volcano plot of DEGs. The *x*-axis represents the log2 FC, and the *y*-axis represents the log10 (*p* value). The blue dots represent downregulated genes, and red dots represent upregulated genes. (b) The Venn diagram shows the overlap between the pink modules of WGCNA and DEGs. (c) Comparison of the expression of four piRNAs in preeclampsia and control group showed that the expression of three piRNAs (uniq_271431, uniq_277797, and piR-hsa-1256314) was statistically different (*p* < 0.05).

We used the limma software package in R to analyze the differential expression of piRNA between PE and the control group. After applying false discovery rate (FDR) < 0.05 and ∣log2 fold change (FC) | ≥2 as the threshold, we obtained 148 differentially expressed piRNA, of which 46 were upregulated and 102 were downregulated (Figure 4(a)). 148 differentially expressed piRNAs were screened from the two groups and compared with the piRNA database (http://www.regulatoryrna.org/database/piRNA/). Among them, 33 are piRNAs that have been found and reported in human genes, and 115 are novel piRNAs that have not been reported. Target piRNAs were identified from the intersection of a Venn diagram between DEGs and the WGCNA pink module (Figure 4(b)). These four overlap piRNAs were considered to be the hub piRNAs, and the sequences of these piRNAs are shown in Table 3.

Then, we used Wilcoxon rank sum test to verify the above four piRNA expression in the two groups of placental tissues again. It was found that the expression difference of the three piRNAs was statistically significant (Figure 4(c)): piR-hsa-1256314, uniq_271431, and uniq_277797. Therefore, we took the above three piRNAs as hub piRNAs for subsequent research.

### 3.5. Prediction and Enrichment Analysis of piRNA Target Genes and Identification of Hub PIWI Protein

**Figure 5:**
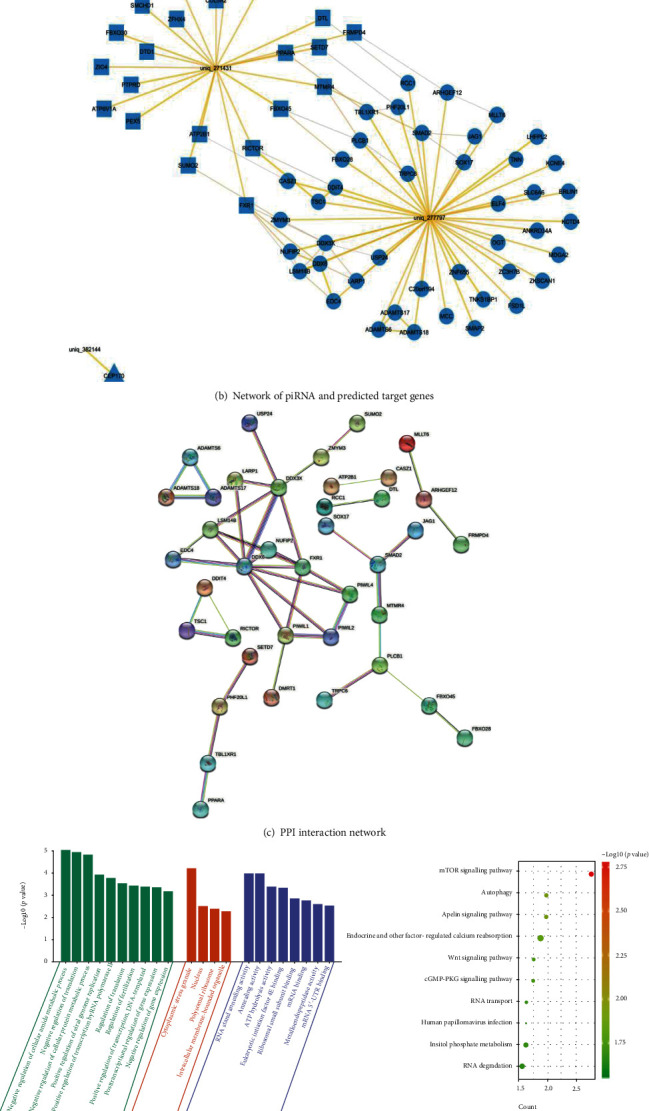
(a) Venn plot of three piRNA target gene prediction databases (TargetScan, miRDB-MicroRNA, and MrmicroT). A total of 66 target genes were found according to the overlap of the three databases (including 22 in uniq_271431, 43 in uniq_277797, and one in piR-hsa-1256314). (b) Network of piRNA and predicted target gene. Blue nodes stand for target genes. The shapes of the nodes represent different piRNAs. The lines represent the regulatory relationship between the piRNAs and target genes. (c) PPI interaction network of target genes determined by STRING. Lines indicate protein-protein interactions. (d) Functional enrichment analysis of target genes. GO enrichment analysis results for target genes. The functional enrichment analysis was performed using the Database for Annotation, Visualization and Integrated Discovery (DAVID). GO terms that are enrichment for target genes are shown in biological processes, molecular function, and cellular component. *y*-axis shows -log10 (*p* value), and *x*-axis shows the terms of GO pathway. The Kyoto Encyclopedia of Genes and Genomes (KEGG) pathways enrichment: the sizes of the circle dots indicate the numbers of enriched genes, and the color of circle indicates the -log10 (*p* value). The *x*-axis represents the number of genes in enrichments. (e) Network centralities calculated by MCC algorithm in CytoHubba plug-in. It can be seen that two of the top three central genes pointed to PIWIL1.

The target genes of three differentially expressed piRNAs were predicted by three tool algorithms of TargetScan, miRDB, and MrmicroT. Their intersection was taken by Venn diagram (Figure 5(a)). A total of 66 target genes were predicted. The network map of piRNAs and their target genes was constructed (Figure 5(b)). The 66 target genes were analyzed by GO (https://david.ncifcrf.gov/home.jsp). It is found that the biological process involved in it is mainly to the negative regulation of cellular amide metabolic process, translation, and cellular protein metabolic process. In KEGG pathway analysis, MTOR signaling path, apelin signaling path, autophagy, Wnt signaling pathway, endocrine, and other factor-regulated calcium reabsorption were found to be the significant pathways in 66 target genes (Figure 5(d)). For further research, we constructed PPI networks between 66 target genes and 4 PIWI genes (PIWIL1, PIWIL2, PIWIL3, and PIWIL4). Then, we used the Cytoscape software to visualize the PPI network. Potential key genes were identified based on maximum centrality (MCC) through the CytoHubba plug-in (Figures 5(b) and 5(c)). It can be seen that two of the top three central genes (FXR1 and DDX6) pointed to PIWIL1. Therefore, among these PIWI family genes, PIWIL1 with the largest number of Hubba nodes was collected for subsequent analysis. We speculated that the regulation of uniq_277797 and uniq_271431 will affect the expression of target genes FXR1 and DDX6, thereby indirectly regulating the expression of the PIWIL1 gene in placental trophoblasts.

### 3.6. PIWIL1 Is Mainly Expressed in the Trophocyte and Is Reduced in Preeclampsia

**Figure 6:**
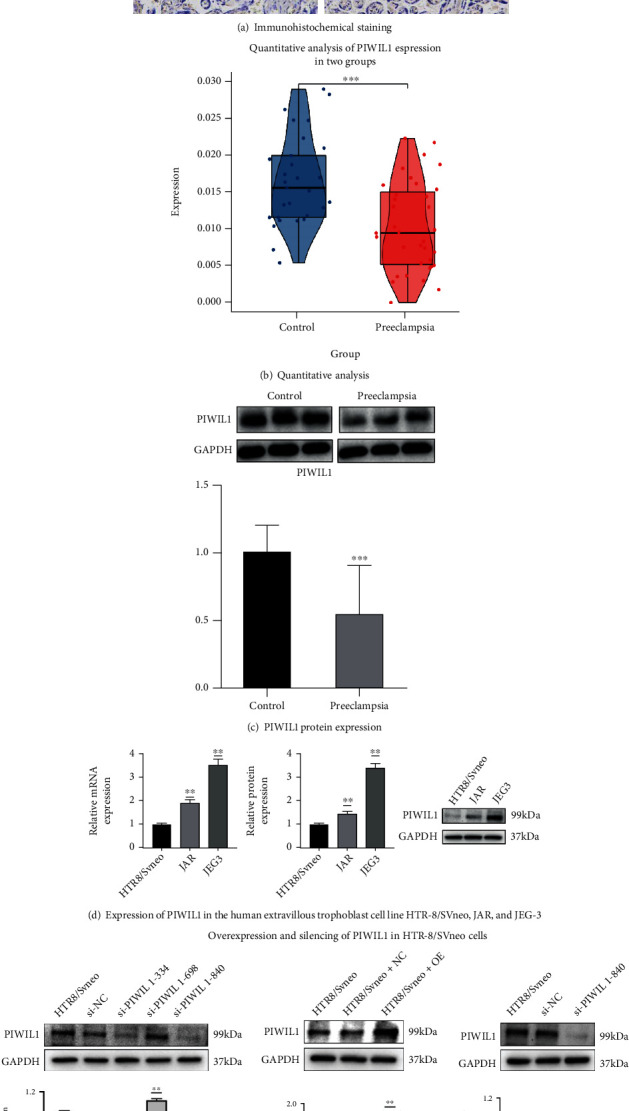
(a) Images of immunohistochemical staining showing the localization of PIWIL1 in placental tissues from normal pregnancies and pregnancies with preeclampsia. (b) Quantitative analysis of PIWIL1 expression in the two groups (35 preeclampsia and 29 normal placentas). (c) PIWIL1 protein expression in placental tissues from normal pregnancies and pregnancies with preeclampsia as visualized by western blotting and quantitative analysis. (d) Expression of PIWIL1 in the human extravillous trophoblast cell line HTR-8/SVneo and choriocarcinoma cell lines JAR and JEG-3. (e) Overexpression and silencing of PIWIL1 in HTR-8/SVneo cells. The figure shows the following: representative PIWIL1 protein bands and quantitative analysis of protein expression in HTR-8/SVneo cells transfected with PIWIL1 siRNAs and a negative control siRNA (NC). PIWIL1 protein bands and quantitative analysis of protein expression in HTR-8/SVneo cells transfected with a PIWIL1-overexpression vector. ^∗^*p* < 0.05, ^∗∗^*p* < 0.01, and ^∗∗∗^*p* < 0.001.

The localization of PIWIL1 in placental tissue was investigated by immunohistochemical staining. The results showed that PIWIL1 protein staining intensity was strongest in the trophocyte, indicating that PIWIL1 is mainly expressed in placental villous trophoblast cells (Figure 6(a)). Quantitative analysis revealed that PIWIL1 expression was significantly lower in placental tissues from pregnancies with preeclampsia than in tissues from normal pregnancies (Figure 6(b)). Western blotting confirmed the decreased expression of PIWIL1 in the placental tissue of pregnancies with preeclampsia (Figure 6(c)).

### 3.7. PIWIL1 Expression Is Associated with Trophoblast Proliferation

To determine whether there is an association between PIWIL1 expression and trophoblast proliferation, PIWIL1 expression was assessed in three cell lines, the trophoblast cell line HTR-8/SVneo and two choriocarcinoma cell lines, JAR and JEG-3. Significantly higher PIWIL1 mRNA and protein expression levels were detected in JAR and JEG-3 cells than in HTR-8/SVneo cells (Figure 6(d)), suggesting that PIWIL1 expression is associated with excessive proliferation of trophoblasts.

### 3.8. Overexpression and Silencing of PIWIL1 in HTR-8/SVneo Cells

To explore the potential effect of PIWIL1 in preeclampsia, PIWIL1 was overexpressed, and its expression was silenced in HTR-8/SVneo cells (Figure 6(e)). Transfection of three tested siRNAs significantly decreased PIWIL1 protein expression, although si-PIWIL1-840-transfected cells showed the lowest PIWIL1 expression levels. Therefore, si-PIWIL1-840 was used to silence PIWIL1 expression, and the results showed PIWIL1 protein expression was reduced in si-PIWIL1-840-transfected HTR-8/SVneo cells when compared with the levels in NC-transfected cells. PIWIL1 protein expression was obviously increased after transfecting a PIWIL1-overexpression vector when compared to the levels in NC-transfected cells. These results indicate that PIWIL1 was successfully overexpressed and silenced in HTR-8/SVneo cells; therefore, these transfected cells could be used in subsequent experiments.

### 3.9. Overexpression of PIWIL1 Promoted the Proliferation and Invasion and Inhibited Apoptosis of HTR-8/SVneo Cells

**Figure 7:**
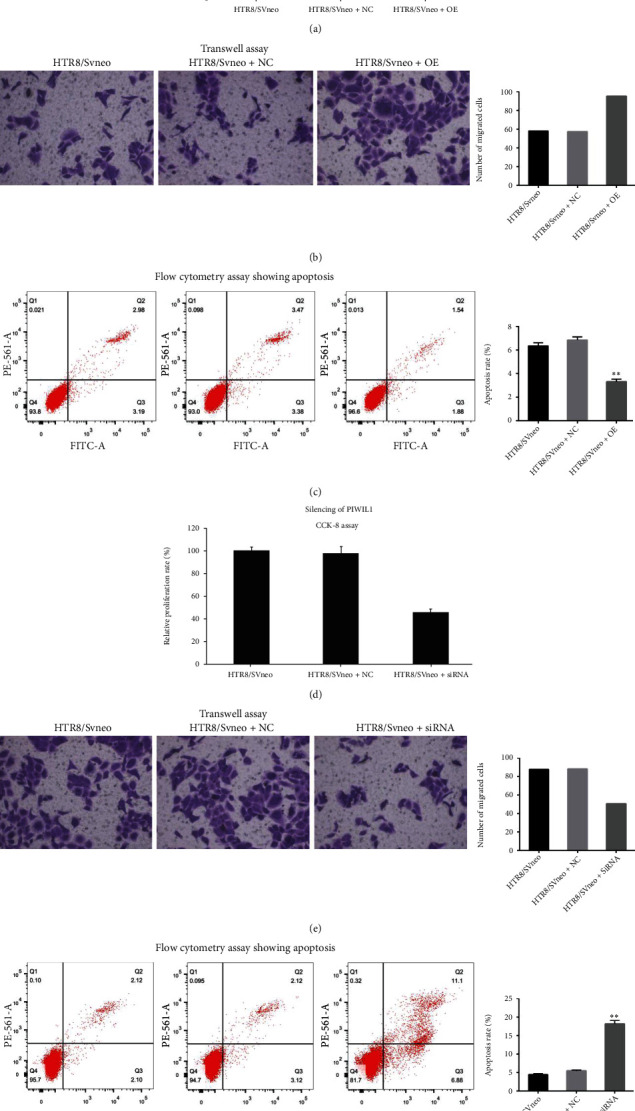
(a–c) Overexpression of PIWIL1 promotes the proliferation and invasion and inhibits apoptosis of HTR-8/SVneo cells. (a) CCK-8 assay showing the relative proliferation rates of HTR-8/SVneo cells with and without PIWIL1 overexpression. (b) Representative images and quantitative analysis of a transwell assay show increased invasion of HTR-8/SVneo cells overexpressing PIWIL1. (c) Representative images and quantitative analysis of a flow cytometry assay showing apoptosis of HTR-8/SVneo cells overexpressing PIWIL1. (d–f) Silencing of PIWIL1 decreases the proliferation and invasion and increases apoptosis of HTR-8/SVneo cells. (d) CCK-8 assay showing the relative proliferation rate of HTR-8/SVneo cells after silencing PIWIL1 expression. (e) Representative images and quantitative analysis of a transwell assay showing the invasion of PIWIL1-silenced HTR-8/SVneo cells. (f) Representative images and quantitative analysis of a flow cytometry assay showing apoptosis of PIWIL1-silenced HTR-8/SVneo cells. ^∗∗^*p* < 0.05 compared with HTR-8/SVneo and HTR-8/SVneo+NC cells.

The effects of PIWIL1 overexpression on cell proliferation were explored. As shown in Figure 7(a), the relative proliferation rate of HTR-8/SVneo cells was remarkably elevated following overexpression of PIWIL1. The results of a transwell assay indicated that the number of invaded cells was markedly higher in PIWIL1-overexpressing HTR-8/SVneo cells than in the other two groups (Figure 7(b)). These results suggest that PIWIL1 overexpression promoted the proliferation and invasion of HTR-8/SVneo cells. Apoptosis was detected using flow cytometry, which showed that PIWIL1 overexpression decreased the apoptosis ratio of HTR-8/SVneo cells (Figure 7(c)).

### 3.10. Silencing of PIWIL1 Decreased the Proliferation and Invasion and Increased Apoptosis of HTR-8/SVneo Cells

The effects of PIWIL1 silencing on cell proliferation, invasion, and apoptosis were investigated by transfecting cells with PIWIL1-siRNA. As expected, cell proliferation and invasion were obviously lower in the HTR-8/SVneo+siRNA group than in the HTR-8/SVneo+NC and HTR-8/SVneo groups (Figures 7(e) and 7(f)), whereas the apoptosis rate of PIWIL1-siRNA-transfected cells was increased compared to the other two groups (Figure 7(g)). These results indicated that silencing of PIWIL1 decreased the proliferation and invasion and increased apoptosis of HTR-8/SVneo cells.

## 4. Discussion

For decades, many researchers in the obstetrics and gynecology field have been working on preeclampsia, but its etiology and pathogenesis have not yet been elucidated. Preeclampsia is considered to be a placenta-derived disorder because it often resolves quickly or spontaneously after delivery of the placenta [17, 18]. Under normal conditions, placental trophoblast invasion presents strict temporal and spatial limitations [19, 20], and many factors located at the maternal-fetal interface regulate this process. Disturbance of any of these factors may cause abnormal trophoblast invasion leading to pregnancy-associated diseases [21, 22]. An overactive trophoblast can lead to placental implantation, staphyloma, and choriocarcinoma. Weak trophoblast function can lead to an inadequate recasting of the spiral arteries of the terminal uterus and pathological obstetrics such as miscarriage, fetal growth restriction, and preeclampsia. Therefore, clarifying the mechanisms regulating the invasion and migration of extravillous trophoblast cells is a breakthrough and a hope for solving these diseases. Thanks to the advances in biotechnology, it has greatly promoted the research on the physical behavior and mechanism of trophoblast. In recent years, many studies have shown that the abnormal expression of some genes is related to the disorder of trophoblast invasion, which may play a role in the pathogenesis of preeclampsia. In recent years, disorders of genes and their products (including proteins and noncoding RNA) have been found in the placenta of patients with preeclampsia, indicating that noncoding RNA plays an important role in regulating cell function [23, 24].

Since the discovery of piRNA, it has been found that it plays a critical biological role in maintaining germline and stem cell function, regulating embryogenesis, maintaining germline DNA integrity, regulating translation and mRNA stability, and regulating epigenetics. piRNA has gradually become a hot spot in the research of noncoding microRNAs. More and more studies have shown that the PIWI/piRNA pathway plays an essential role in tumorigenesis and development [25, 26]. As a new member in the field of noncoding RNA, the relationship between piRNA and abnormal trophoblast function in preeclampsia is worth exploring. Chirn et al. [27] found a unique set of piRNA cluster loci, Eutherian-Conserved piRNA cluster (ECpiC) locus, in the development spectrum of mammals. This locus produced a rich piRNA antisense to the stox1 transcript. The STOX1 gene has been shown to cause placental dysfunction, which may be the center of the common pathway leading to preeclampsia [28, 29]. Therefore, we speculate that piRNA may play a potentially important role in the pathogenesis of preeclampsia. Considering that there are few studies on PIWI/piRNA in the pathogenesis of preeclampsia, we want to understand the role of the PIWI/piRNA pathway in the pathogenesis of preeclampsia, its impact on the proliferation and invasion of placental trophoblast, and which piRNAs may be involved in the occurrence of the disease. At present, more than 3000 piRNAs have been confirmed in humans, and there are still many piRNAs that have not been found and verified [30]. In this study, we found two differentially expressed novel piRNAs, uniq_277797 and uniq_271431. Through bioinformatics analysis, we hypothesized that they may affect the expression of target genes FXR1 and DDX6, thereby indirectly regulating the expression of the PIWIL1 gene in the placental trophoblasts.

PIWI proteins, which were first reported due to their evolutionarily conserved functions in regulating the self-renewal of germline stem cells, function mainly by binding to piRNAs that are 24–32 nucleotides in length [31, 32]. In humans, there are four PIWI proteins, PIWIL1–4 [33]. Several studies have been focused on the expression of PIWIL1 and its roles in tumors [34, 35]. PIWIL1 expression status has been proposed as a prognostic biomarker in many tumors, such as pancreatic cancer [36], colorectal cancer [15], and colonic adenoma and adenocarcinoma [37]. In addition, PIWIL1 has also been reported to regulate genes implicated in cell proliferation, migration, and apoptosis as well as the cell cycle [38, 39]. Leavey et al. [40] collected a microarray dataset containing 330 placentas (157 PE and 173 non-PE). Among 14,653 genes, 3,663 genes with the highest (top quartile) variance were found for clustering. PIWIL1 is one of the differentially expressed genes. To the best of our knowledge, our study is the first to investigate the role of PIWIL1 on placental trophoblast invasiveness in preeclampsia. We observed that PIWIL1 was mainly expressed in placental villous trophoblast cells, and its expression was reduced in pregnancies with preeclampsia. PIWIL1 expression was strongly correlated with excessive cell proliferation of trophoblasts. Overexpression of PIWIL1 promoted cell proliferation and invasion and inhibited apoptosis of HTR-8/SVneo cells. These effects could be reversed by silencing PIWIL1. These results emphasize the important role of PIWIL1 in preeclampsia. Based on the most representative “two-stage disease” theory, the pathological and physiological changes in preeclampsia are caused by shallow invasion of trophoblasts, leading to poor remodeling of the spiral arteries [8, 41]. The current study showed that PIWIL1 was mainly expressed in the placental trophoblast cells, and that PIWIL1 expression was associated with excessive trophoblast cell proliferation. This emphasized the close association of PIWIL1 expression with placental trophoblast cells. Placental trophoblast cells are responsible for the placental invasion and uterine vessel remodeling [42, 43]. Although preeclampsia develops typically after 20 weeks of gestation, abnormal placental trophoblast function may start at the beginning of gestation and continue until delivery of the placenta at the end of gestation [44]. Therefore, uncovering the factors regulating the biological functions of placental trophoblast cells should improve our understanding of the pathogenesis of preeclampsia.

HTR-8/SVneo cells were chosen to study trophoblast functional because this cell line has been widely used in studies on the pathogenesis of preeclampsia [45, 46]. A previous study reported that apoptosis of placental trophoblast cells from pregnant women with preeclampsia was significantly increased, and that the degree of apoptosis was correlated with disease severity [47]. PIWIL1 was overexpressed or silenced in HTR-8/SVneo trophoblast cells, and the results revealed that overexpression of PIWIL1 facilitated cell proliferation and invasion and inhibited apoptosis, and these effects were reversed by silencing of PIWIL1. Additionally, immunohistochemical staining indicated that PIWIL1 expression was markedly decreased in the placental villous trophoblasts of patients with preeclampsia. These results suggested that reduced expression of PIWIL1 leads to decreases in the proliferation and invasion of placental trophoblast in preeclampsia. Therefore, we speculated that PIWIL1 might be involved in the pathogenesis of preeclampsia by repressing the proliferation and invasion of trophoblasts, resulting in shallow implantation of the placenta and poor remodeling of the spiral arteries. This is consistent with the claim that placental trophoblast cells from patients with preeclampsia showed reduced viability and invasion ability [48].

In conclusion, this study showed that PIWIL1 is mainly expressed in placental trophoblast cells and PIWIL1 expression was strongly correlated with proliferation and invasiveness of trophoblast cells. PIWIL1 expression was reduced in patients with preeclampsia, and silencing of PIWIL1 expression inhibited the proliferation and invasion of trophoblasts and promoted the apoptosis of placental trophoblast cells. We hypothesized that piRNA regulation would affect the expression of target genes, thereby indirectly regulating the expression of the PIWIL1 gene in placental trophoblast cells, resulting in shallow placental implantation and the occurrence of preeclampsia. Therefore, PIWIL1/piRNA pathway may be a potential target in preeclampsia in the future.

## Figures and Tables

**Table 1:** Sequences of small interfering RNA targeting PIWIL1.

Names	Sequence (5′ to 3′)
NM_004764_stealth_344	CACTGCCAGTCAGCAACCTGGTTAT
NM_004764_stealth_698	CAGAAGACTCCGTTCAGCTCTTCTT
NM_004764_stealth_840	GATGTGAGGATAACGATCACTTTAA
siRNA_NC	CACGACCGACTCAACGGTCTGTTAT

**Table 2:** Clinical characteristics of the included subjects.

	Normal (*n* = 34)	Preeclampsia (*n* = 40)	*p* value
Age (years)	30.2 ± 3.3	31.9 ± 4.8	0.147
Gravidity (times)	1.4 ± 0.7	1.8 ± 0.2	0.090
Parity (times)	1.0 ± 0.1	1.2 ± 0.3	0.032
Delivery gestational week (weeks)	39.4 ± 1.05	35.4 ± 2.9	0.001
Birth weight (g)	3375.4 ± 297.4	2358.7 ± 697.9	0.001
Apgar score	9.9 ± 0.3	9.1 ± 1.4	0.009
Postpartum hemorrhage (ml)	223.6 ± 54.1	327.2 ± 91.8	0.001

**Table 3:** Differentially expressed piRNAs obtained by WGCNA and DEGs.

piRNA ID	Sequence	Regulation	Fold change	*p* value
uniq_254024	GACGGTGAATACAGGTCCGGAAGTCTGAGGTC	Down	-2.302	0.046
uniq_271431	GTCAGGATGGCCGAGTGGTCTAAGGC	Up	2.151	0.031
uniq_277797	TCAAGTGGTGTCATCTTACTACTGAG	Up	3.111	0.016
piR-hsa-1256314	GATAAGGATTGGCTCTAAGGGCTGGGG	Up	2.766	0.028

## Data Availability

All the data are available upon request.
